# Clinical Significance of 18F-Fluorodeoxyglucose Avid Prostate Gland Incidentalomas on Positron Emission Tomography/Computed Tomography

**DOI:** 10.4274/mirt.07769

**Published:** 2017-06-01

**Authors:** William Makis, Anthony Ciarallo

**Affiliations:** 1 Cross Cancer Institute, Department of Diagnostic Imaging, Edmonton, Canada; 2 McGill University Health Centre Glen Site, Department of Nuclear Medicine, Montreal, Canada

**Keywords:** Prostate incidentaloma, prostate carcinoma, 18F-fluorodeoxyglucose, positron emission tomography/computed tomography, prostate specific antigen

## Abstract

**Objective:**

The aim of this study was to evaluate the clinical significance of incidental focal uptake of ^18^F-fluorodeoxyglucose (^18^F-FDG) on positron emission tomography/computed tomography (PET/CT) in the prostate glands of cancer patients.

**Methods:**

A retrospective review of 3122 consecutive male patients who underwent ^18^F-FDG PET/CT studies with an oncologic indication, over the course of four years, was performed. Studies with incidental ^18^F-FDG uptake in the prostate gland were further analyzed.

**Results:**

Incidental ^18^F-FDG uptake in the prostate gland was identified in 65/3122 men (2.1%). Sufficient follow-up data (≥12 months) were available in 53 patients, of whom 11 had a biopsy and 42 had clinical and imaging follow-up. Malignancy was histologically diagnosed in 4 out of 53 patients (7.5%). There was no statistically significant difference in ^18^F-FDG uptake values between benign prostate lesions [maximum standardized uptake value (SUV_max_) 7.3] and malignant ones (SUV_max_ 7.2, p=0.95). There was a statistically significant difference between the serum prostate specific antigen (PSA) of the benign group (n=24, PSA=2.7 ng/mL) and the malignant group (n=4, PSA=9.2 ng/mL, p<0.001). There was a direct correlation between SUV_max_ and Gleason score.

**Conclusion:**

^18^F-FDG positive prostate incidentalomas were detected in 2.1% of oncologic PET/CT scans and of these 7.5% were malignant. SUV_max_ was not useful for distinguishing between benign and malignant incidental prostate lesions. ^18^F-FDG avid prostate incidentalomas on PET/CT should prompt a recommendation for obtaining a serum PSA and further investigation if serum PSA is elevated.

## Abstract (tr)

**Amaç:**

Bu çalışmanın amacı kanser hastalarının prostat bezinde pozitron emisyon tomografisi/bilgisayarlı tomografide (PET/BT) insidental olarak saptanan fokal ^18^F-fluorodeoksiglukoz (^18^F-FDG) tutulumunun klinik önemini değerlendirmektir.

**Yöntem:**

Dört yıllık bir dönemde onkolojik nedenlerle ^18^F-FDG-PET/BT uygulanmış 3122 ardışık erkek hasta retrospektif olarak incelendi. Prostat bezinde insidental ^18^F-FDG tutulumu saptanan hastalar detaylı olarak değerlendirildi.

**Bulgular:**

Prostat bezinde insidental ^18^F-FDG tutulumu 65/3,122 erkekte (%2,1) saptandı. Yeterli takip verisi (≥12 ay) olan 53 hasta mevcuttu, bunların 11’ine biyopsi uygulanmış 42’si klinik ve radyolojik olarak takip edilmişti. Elli üç hastanın dördünde malignite histolojik olarak saptanmıştı (%7,5). Benign ve malign prostat lezyonlarında ^18^F-FDG tutulum değerleri açısından istatistik olarak anlamlı fark yoktu [maksimum standart uptake değeri (SUV_maks_) benign: SUV_maks_ 7,3, malign: SUV_maks_ 7,2, p=0,95]. Benign ve malign grup hastalarda serum prostat spesifik antijen (PSA) değerleri arasında istatistiksel olarak anlamlı fark vardı (benign grup n=24, PSA=2,7 ng/mL, malign grup n=4, PSA=9,2 ng/mL, p<0,001). SUVmax ve Gleason skoru arasında direkt korelasyon mevcuttu.

**Sonuç:**

Onkolojik PET/BT görüntülemelerinin %2,1’inde ^18^F-FDG pozitif prostat bezi insidentaloması saptandı ve bunların %7,5’i malign idi. SUV_maks_, benign ve malign insidental prostat lezyonlarını ayırt etmede yararlı değildi. PET/BT’de ^18^F-FDG tutulumu olan prostat bezi insidentaloması varlığında serum PSA değerinin bakılması önerilmeli ve eğer PSA yüksek ise ileri tetkik gerekliliği akılda tutulmalıdır.

## INTRODUCTION

Since positron emission tomography/computed tomography (PET/CT) was first introduced for the staging and follow-up of various malignancies, PET/CT readers have been faced with the challenge of interpreting foci of increased ^18^F-fluorodeoxyglucose (^18^F-FDG) uptake in unexpected locations. In addition to malignancy, ^18^F-FDG uptake has been described in various sites of normal physiologic processes and tracer biodistribution, in benign nodules and masses, and in infectious and inflammatory processes (1,2,3). Increased ^18^F-FDG activity in locations not typical for metastatic spread in patients known for malignancy may alternatively represent an unrelated benign process or even a second primary malignancy, thus complicating the interpretation of the PET/CT study. The most common locations of potentially malignant incidental ^18^F-FDG uptake reported in the literature include: breast, gastrointestinal system, the prostate, thyroid, adrenal and parotid glands (4,5,6,7,8,9,10). Locations such as the thyroid, adrenal, and gastrointestinal system have been investigated extensively in the literature, while locations such as the prostate gland continue to confound PET/CT readers.

Several studies have investigated the clinical significance of ^18^F-FDG positive prostate incidentalomas (11,12,13,14,15,16,17). The aim of this study was to determine the frequency of unexpected focal uptake of ^18^F-FDG on PET/CT in the prostate glands of cancer patients and to detect the proportion of malignant cases within this group. We examined the possibility of using standardized uptake value (SUV_max_) to differentiate benign causes of incidental prostate ^18^F-FDG uptake from malignant ones. We also examined if serum prostate specific antigen (PSA) values were different in the benign group as compared to the malignant group.

## MATERIALS AND METHODS

### Study Design and Patient Population

3122 consecutive male patients who underwent ^18^F-FDG PET/CT studies with an oncologic indication over the course of 48 months (from January 1, 2006 to December 31, 2009) were retrospectively reviewed at our institution, a tertiary care academic hospital. The PET/CT reports that made a special reference to focal ^18^F-FDG uptake in the prostate gland provided the basis for this study.

Sixty-five patients had incidental ^18^F-FDG uptake in the prostate gland and represented the study group. Patients with a previous prostate malignancy or prostatectomy (n=3) were excluded from the study. Patients with insufficient follow-up data (<12 months) (n=9) were also excluded from this study. The remaining 53 cases constituted the study group for further assessment of clinical significance of incidental ^18^F-FDG uptake in the prostate gland.

### Positron Emission Tomography/Computed Tomography Imaging

^18^F-FDG PET/CT studies were performed on a hybrid PET/CT scanner (Discovery ST, General Electric Medical Systems, Waukesha, WI, USA), which combines a dedicated, full-ring PET scanner with a 16-slice CT scanner. Patients were required to fast for at least 6 hours before the time of their appointment, and waited in a quiet dark room the morning of their scan. Blood glucose levels were recorded immediately prior to ^18^F-FDG administration. If the serum glucose level was greater than 11.1 mmol/L (200 mg/dL), the study was rescheduled. A volume of 400 mL of barium sulfate oral contrast was administered, and 8.14 MBq/kg of ^18^F-FDG was injected intravenously up to a maximum dose of 740 MBq. Approximately sixty minutes following ^18^F-FDG injection, CT and PET images were consecutively acquired from the base of the skull to the upper thighs, with additional images of the extremities acquired if needed. CT scan settings were: 140 kVp, 90-110 mA (depending on the body weight), a rotation time of 0.8 s, a table speed of 17 mm per gantry rotation, a pitch of 1.75:1, and a detector row configuration of 16×0.625 mm. For the PET portion of the study, a 2-D acquisition was performed and images were acquired for 4-5 min per bed position (depending on the body weight) up to 5 to 6 total bed positions (depending on the patient’s height). The patient was allowed to breathe normally during the PET and CT acquisitions.

Data obtained from the CT acquisition were used for attenuation correction and fusion with PET images. The PET data were reconstructed iteratively using ordered subset expectation maximization software provided by the manufacturer (21 subsets, 2 iterations). PET attenuation corrected, PET non-attenuation corrected, CT, and PET/CT fusion images of the whole body were displayed in the transaxial, coronal, and sagittal planes and were reviewed on a dedicated workstation (Xeleris 2.0, GE Healthcare, Waukesha, WI, USA). PET data were also displayed in a rotating maximum intensity projection image.

### Interpretation and Analysis of Positron Emission Tomography/Computed Tomography Images

All PET/CT images were interpreted using visualization and semi-quantitative analysis (SUV_max_ corrected for body weight) by two experienced nuclear physicians, independently. Any focal ^18^F-FDG uptake in the prostate gland was noted and each nuclear medicine physician measured the SUV_max_ corrected for body weight, using a spherical region of interest at the site of the most intense uptake in the prostate gland.

### Diagnosis and Follow-up

Final diagnosis of benign or malignant prostate incidentaloma was based on histologic tissue sampling in 11 of 53 patients. The remaining 42 patients were assessed clinically and/or by serial imaging with magnetic resonance imaging (MRI) or PET/CT. Lesions were considered benign on serial imaging if there was no further evidence of malignancy in the prostate gland or if there was evidence of regression in SUV_max_ by at least 50% in the absence of treatment over a period of at least 12 months. Serum PSA values obtained within 6 months of the PET/CT scan were compared in the benign and malignant groups.

### Statistical Analysis

The Mann-Whitney U test was used to determine if there was a significant difference between the mean SUV_max_ values of the benign and malignant groups. The Mann-Whitney U (two tailed) test was used to detect a significant difference between the serum PSA values of the benign and malignant groups. A p value less than 0.05 was considered to indicate a statistically significant difference. A correlation between Gleason score and SUV_max_ was established using the Spearman’s rank correlation coefficient.

## RESULTS

The mean age of the study group was 69.3 years (range: 45-87 years). The primary malignant tumors of the cohort and their relative distribution are listed below (Table 1). Incidental focal ^18^F-FDG uptake in the prostate gland was found in 65 of 3122 (2.1%) men scanned consecutively with PET/CT for an oncologic indication. The mean age of patients with benign prostate lesions was 68.8 years as compared to 74.0 years in patients with prostate malignancy. The distribution of abnormal ^18^F-FDG prostate uptake in the 53 patients with sufficient follow-up were identified as: peripheral n=37 (69.8%), central n=7 (13.2%), and multifocal or heterogeneous n=9 (17.0%) (Table 2).

Histologic tissue sampling was available in 11 of 53 patients, and the remaining 42 patients were assessed clinically and/or by serial imaging with MRI or PET/CT. The mean clinical follow-up period of these 42 patients was 33 months (range: 12-66 months). Out of 53 patients, 49 (92.5%) were diagnosed with a benign prostate process and 4 (7.5%) were diagnosed with prostate adenocarcinoma. There was no statistically significant difference in terms of the mean SUV_max_ values between the benign (SUV_max_ 7.3) and the malignant group (SUV_max_ 7.2) (p value 0.95, Mann-Whitney U test) (Table 3). The four malignant prostate incidentalomas were identified in patients with various primary malignancies and all four malignant cases had ^18^F-FDG uptake in the peripheral zone of the prostate gland (Table 4). The SUV_max_ range of the four malignant cases was 4.7-9.9, while the SUV_max_ range of the seven benign biopsied cases was 2.1-22.0, with histology evaluation showing two cases of benign prostatic hyperplasia (BPH) and five cases of benign prostate tissue (Table 5).

**Figure 1:**
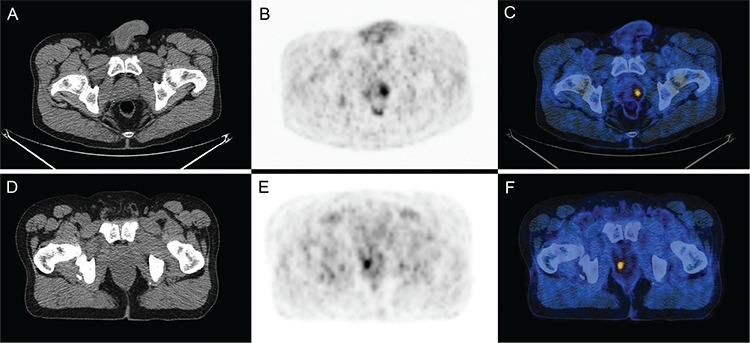
Two histologically confirmed cases of ^18^F-fluorodeoxyglucose (^18^F-FDG) positive prostate incidentaloma, one benign and one malignant, with similar maximum standardized uptake value (SUV_max_) values. A 58 year old man with previous history of Hodgkin lymphoma (Table 5, patient 8) had a follow-up ^18^F-FDG positron emission tomography/computed tomography (PET/CT). A serum prostate specific antigen done within 6 months of the PET/CT was 2.0 ng/mL and a biopsy of the prostate did not reveal any malignancy, only benign prostate tissue. A, B, C) Axial PET/CT images show a focus of intense ^18^F-FDG uptake in the prostate with SUV_max_ 4.4, consistent with a false positive. A 77 year old man with a previous history of rectal cancer (Table 4, patient 4) had a follow-up ^18^F-FDG-PET/CT. A serum prostate specific antigen done within 1 month was 8.9 ng/mL and biopsy of the prostate showed prostate carcinoma with Gleason score 6 (3+3). D, E, F) Axial PET/CT images show a focus of ^18^F-FDG uptake in the prostate with SUV_max_ 4.7, consistent with a true positive

Serum PSA values obtained within 6 months of the PET/CT were available in 28 of 53 (52.8%) patients. Of those patients with available serum PSA values, 24 of 28 (85.7%) were diagnosed as benign and 4 of 28 (14.3%) were diagnosed as malignant. Mean serum PSA value of patients with a benign prostate process was 2.7 ng/mL versus 9.2 ng/mL for patients with a malignant prostate incidentaloma, a statistically significant difference with a p value <0.001 by Mann-Whitney U test (Figure 1). The lowest PSA value of the malignant cases was 6.7 ng/mL.

**Figure 2:**
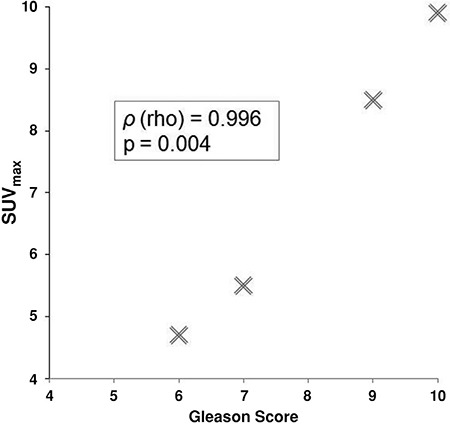
The Gleason scores of the malignant prostate incidentalomas correlated directly with maximum standardized uptake value (Spearman’s rank coefficient, rho=0.996, p=0.004) *SUV_max_: Maximum standardized uptake value*

The Gleason scores of the malignant prostate incidentalomas correlated directly with SUV_max_ (Spearman’s rank coefficient, rho=0.996, p=0.004) (Figure 2).

## DISCUSSION

In our study, 2.1% of PET/CT scans performed in men with an oncologic indication revealed incidental uptake in the prostate gland, in keeping with previously reported values ranging from 0.6% to 4.5% (11,12,13,14,15,16,17). However, the incidence of ^18^F-FDG positive prostate incidentalomas that have been confirmed to be malignant varies widely in the literature, from 5.4% (11) up to 58.0% (13), making it difficult for the interpreting physician to decide what to report or recommend when faced with this uncommon incidental PET/CT finding. In our patient population, 7.5% (4 of 53) of ^18^F-FDG positive prostate incidentalomas were malignant. There was no particular cancer population which seemed to be at a higher risk of incidental prostate cancer. These findings are similar to those reported by Han et al. (11), who reported prostate malignancy in 5.4% of 55 incidentalomas. It is important to note that in most prostate incidentaloma papers, a significant number of prostate incidentalomas have not been investigated further and do not have any long term follow-up, likely resulting in an overestimation of reported malignancy rates. Only studies by Han et al. (11) and Seino et al. (15) evaluated most of their prostate incidentalomas [87% 55/63 by Han et al. (11), 92% 49/53 by Seino et al. (15)]. Neither of these studies had long term follow-up of their benign prostate incidentalomas. Our mean follow-up period for benign prostate incidentalomas was 33 months.

All prostate incidentaloma studies published thus far confirm that quantitative analysis using SUV_max_ values alone cannot differentiate benign incidental prostate lesions from malignant ones. Similarly, our data failed to demonstrate a statistically significant difference between mean SUV_max_ values for the benign and malignant groups. High SUV_max_ values have been reported in several benign prostate conditions such as prostatitis (18), BPH (19), as well as other malignant prostate conditions (20,21,22,23), such as seminoma (20), sarcomatoid carcinoma (21), and neuroendocrine tumor of the prostate (22). If clinical significance of an ^18^F-FDG positive prostate incidentaloma is to be determined, it requires more information than SUV_max_ alone.

Prostate cancer is often confirmed by histological examination of a sample obtained by needle biopsy. However, this intervention is invasive and unnecessary in the vast majority of patients with ^18^F-FDG positive prostate incidentalomas. PSA and digital rectal examination are useful non-invasive screening tests routinely used in clinical practice (24,25,26,27,28,29,30). In our population, there was a statistically significant difference between the serum PSA values of benign prostate incidentalomas (n=24, PSA=2.7 ng/mL) and malignant prostate incidentalomas (n=4, PSA=9.2 ng/mL, p<0.001), which is in keeping with the majority of published studies on ^18^F-FDG avid prostate incidentalomas (11,12,13,14,15,16,17).

Some investigators have noted a statistically significant association between SUV_max_ and Gleason score, whereby prostate lesions with higher Gleason scores also had higher SUV_max_ values on PET/CT (31,32). Our study found a direct correlation between Gleason score and SUV_max_ in malignant prostate incidentaloma cases. Even though most ^18^F-FDG positive prostate incidentalomas are statistically benign, a markedly elevated SUV_max_ arguably warrants closer follow up in these patients to avoid missing an aggressive malignancy.

There are several limitations to our study. Although the minimum follow-up time was set at 12 months, and the mean period of clinical follow-up of 42 prostate incidentalomas who did not have a biopsy was 33 months, longer follow-up would likely improve the results, especially due to the indolent nature of prostate cancer. Another limitation was that serum PSA values were not obtained in all prostate incidentaloma patients, and the timing of obtained PSA values ranged from the same day of to up to 6 months within the PET/CT. Ideally, serum PSA values should be available in all patients with prostate incidentalomas and performed at the same time as the PET/CT.

Several PSA related indices, such as free-to-total PSA ratio (F/T ratio), PSA density (PSAD) and PSA transition zone density (PSATZ) could further improve the differentiation of benign ^18^F-FDG positive prostate incidentalomas from malignant ones. These indices appear to improve cancer detection sensitivity and specificity in patients with low serum PSA levels. The ratio of free-to-total PSA (F/T ratio) is known to be reduced in cases of prostate cancer. For patients with PSA levels between 4.0 and 10.0 ng/mL, the recommended cut-off value for F/T is ≤0.25. The ideal cut-off for PSAD is 0.15 ng/mL/cm^3^ (26,27,28,29,30). These indices may one day play a role in helping determine whether a patient with an ^18^F-FDG positive prostate incidentaloma is at high risk for harboring a prostate malignancy and should have a biopsy or whether a biopsy is not necessary. Novel PET/CT agents such as gallium-68 (68Ga)- prostate-specific membrane antigen (PSMA) may also become useful in differentiating benign ^18^F-FDG avid prostate incidentalomas from malignant ones, as several studies have recently reported prostate cancer detection rates by 68Ga-PSMA PET/CT imaging in the range of 90-100% (33,34,35).

## CONCLUSION

In our patient population, 2.1% of ^18^F-FDG PET/CT scans performed in men for an oncologic indication revealed incidental ^18^F-FDG uptake in the prostate gland. Among those prostate incidentalomas, 7.5% were malignant. SUV_max_ alone was unable to differentiate between benign and malignant prostate lesions, however there was a statistically significant difference between the serum PSA of benign and malignant prostate lesions. These findings suggest that obtaining a serum PSA level in a patient with an ^18^F-FDG positive prostate incidentaloma is a reasonable initial course of action. Patients with significantly elevated serum PSA levels can then be investigated further with biopsy, or followed non-invasively with serial PSAs, clinical examination or follow-up imaging.

## Figures and Tables

**Table 1:**
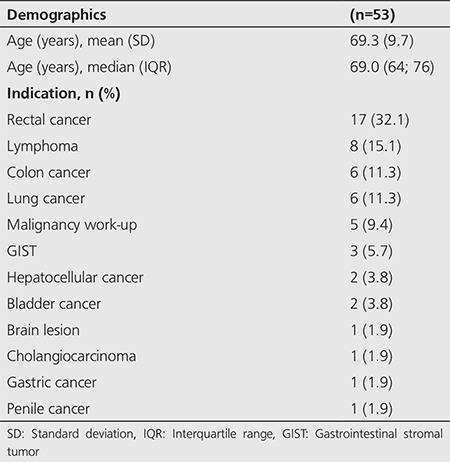
Characteristics of the cohort

**Table 2:**
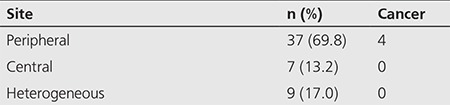
Distribution of prostate 18F-fluorodeoxyglucose uptake

**Table 3:**
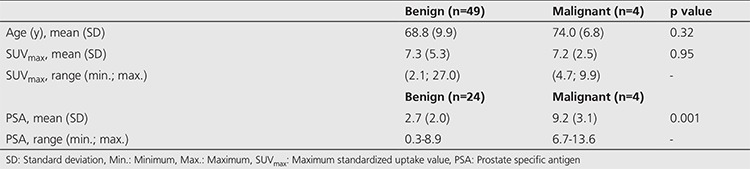
Benign vs. malignant prostate lesions

**Table 4:**
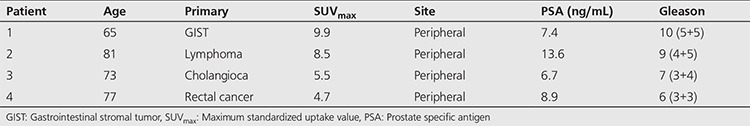
Biopsied malignant prostate incidentalomas

**Table 5:**
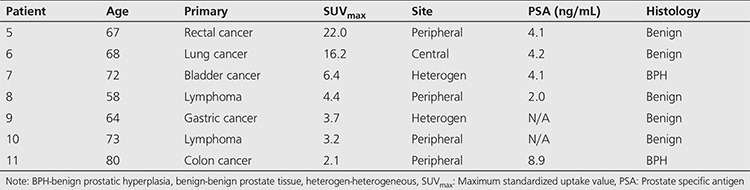
Biopsied benign prostate incidentalomas
